# Association of *PON2* Gene Polymorphisms (Ser311Cys and Ala148Gly) With the Risk of Developing Type 2 Diabetes Mellitus in the Chinese Population

**DOI:** 10.3389/fendo.2018.00495

**Published:** 2018-08-27

**Authors:** Huan Ren, Sheng-Lan Tan, Mou-Ze Liu, Hoan L. Banh, Jian-Quan Luo

**Affiliations:** ^1^Department of Pharmacy, The Second Xiangya Hospital, Central South University, Changsha, China; ^2^Institute of Clinical Pharmacy, Central South University, Changsha, China; ^3^Department of Clinical Pharmacology, Xiangya Hospital, Central South University, Changsha, China; ^4^Department of Family Medicine, Faculty of Medicine and Dentistry, University of Alberta, Edmonton, AB, Canada

**Keywords:** *PON2*, gene polymorphism, Ser311Cys, Ala148Gly, susceptibility, type 2 diabetes

## Abstract

**Background:** The association between paraoxonase 2 (PON2) gene polymorphisms and type 2 diabetes mellitus (T2DM) has been extensively investigated in the Chinese population with conflicting results. In this study, we systematically evaluated the association between *PON2* Ser311Cys and Ala148Gly polymorphisms and T2DM risk by pooling all relevant studies.

**Methods:** We searched PubMed, Embase, CNKI, and Wanfang databases for the studies. The strength of association was determined by the allelic, homozygous, heterozygous, recessive, and dominant genetic models and measured as odds ratio (OR) and 95% confidence interval (CI), under fixed- or random-effect models.

**Results:** There was no significant association between *PON2* Ser311Cys polymorphism and T2DM under any of the genetic models: allelic (OR = 1.06, 95% CI = 0.77–1.45; *P* = 0.721), heterozygous (OR = 1.13, 95% CI = 0.87–1.45; *P* = 0.362), dominant (OR = 1.10, 95% CI = 0.80–1.51; *P* = 0.562), recessive (OR = 0.87, 95% CI = 0.48–1.58; *P* = 0.648), homozygous (OR = 0.94, 95% CI = 0.47–1.89; *P* = 0.865). Similarly, no significant association was found in *PON2* Arg148Gly polymorphism under any of the models: allelic (OR = 1.17, 95% CI = 0.91–1.50; *P* = 0.218), heterozygous (OR = 1.28, 95% CI = 0.94–1.74; *P* = 0.117), dominant (OR = 1.25, 95% CI = 0.93–1.67; *P* = 0.142), recessive (OR = 0.99, 95% CI = 0.52–1.88; *P* = 0.973), homozygous (OR = 1.08, 95% CI = 0.57–2.07; *P* = 0.808).

**Conclusions:** The *PON2* Ser311Cys and Ala148Gly polymorphisms were not associated with the risk of developing T2DM in the Chinese population.

## Introduction

Diabetes is a major cause of mortality and morbidity worldwide, and it has become an important public health problem in China (1, 2). National surveys indicate that the prevalence of diabetes increased dramatically among Chinese adults during the past three decades. The rising prevalence of diabetes highlights the urgent need for aggressive strategies aimed at the prevention and control of diabetes (3, 4). Type 2 diabetes mellitus (T2DM) is the most prevalent type of diabetes around the world. Substantial evidence demonstrates that T2DM is a complex metabolic disease triggered by lifestyle, environmental and genetic factors (5–8).

The paraoxonase 2 (PON2) gene encodes a member of the PON multigene family, which includes two other known members sharing approximately 65% sequence similarity at the amino acid level, PON1 and PON3, located adjacent to each other on the chromosome 7q21.3–22.1 in humans (9). PON1 and PON3 are primarily expressed in the liver while PON2 is ubiquitously expressed in many different mammalian tissues (10). The *PON2* gene contains eight introns and nine exons. It is polymorphic and several common single nucleotide polymorphisms (SNP) have been identified thus far. Currently, genetic variations in *PON2* gene may be associated with a number of disorders, such as cardiovascular disease and T2DM (11–14).

In the previous genetic epidemiologic studies, the association between the Ser311Cys (rs6954345/rs7493) and Ala148Gly (rs11545942/rs12026) polymorphisms in *PON2* gene and the risk in developing T2DM has increased the focus on the Chinese population. In Qu's study, *PON2* Ser311Cys gene polymorphism was found to be significantly associated with an increased risk of T2DM in a northern Chinese population (15). In 2014, Xu and Dai also revealed that *PON2* 311Cys allele could increase the T2DM risk in the Qinghai population (16). In contrast, Sun reported an opposite result that the *PON2* 311Ser contributed to the development of T2DM in another northern Chinese population, with the frequency of *PON2* 311Ser allele being significantly higher in T2DM patients than the control groups (17). In addition, Xu et al. (18) failed to find an association between *PON2* Ser311Cys gene variation and T2DM in an Anhui population in 2007 and Sun et al. (19) also observed a similar effect in another northern population.

It is crucial to address this inconsistency among the currently published studies. In the present study, a comprehensive meta-analysis was conducted by pooling all qualified individual data from case-control studies to make a precise conclusion on the association between *PON2* Ser311Cys and Ala148Gly gene polymorphisms and the risk of developing T2DM in the Chinese population.

## Methods

This study was performed in accordance with the guidelines of the Meta-analysis of Observational Studies in Epidemiology (MOOSE) statement (20).

### Publication search and inclusion criteria

The following electronic databases PubMed, Embase, Wanfang Data, and China National Knowledge Infrastructure (CNKI) were searched for all case-control studies published up to August 2017 on the association of *PON2* Ser311Cys and Ala148Gly gene polymorphisms and the risk of developing T2DM in the Chinese population. The search was performed in the databases in English and Chinese with proper keywords: paraoxonase 2 gene (“paraoxonase 2,” “*PON2*”) or variations (e.g., “Single Nucleotide Polymorphism,” “SNP,” “polymorphism,” “Mutation,” “variation,” “variant”) in combination with T2DM (e.g., “Type 2 Diabetes Mellitus,” “Diabetes Mellitus, Type 2,” “Noninsulin Dependent Diabetes Mellitus,” “Diabetes Mellitus, Noninsulin Dependent”). In addition, the references from relevant reviews or primary studies were hand searched to identify additional studies.

Two investigators independently reviewed all studies for eligibility. The inclusion criteria were as follows: (a) study investigating the associations between Ser311Cys (rs6954345/rs7493) and Ala148Gly (rs11545942/rs12026) in *PON2* gene and the risk of developing T2DM. (b) case-control studies, regardless of sample size. (c) studies providing the numbers of *PON2* genotypes or alleles in case and control subjects. (d) the distribution of genotypes in the control groups met the Hardy-Weinberg equilibrium (HWE, *P* > 0.05). Disagreement between two investigators regarding the eligibility of any study was resolved by the consensus of a third reviewer.

### Data extraction

Data extraction was independently performed by two reviewers. A consensus on all items of data extraction was reached by both reviewers. The following items were extracted from each study: the first author's name, publication year, region of the study, mean age and gender distribution of the participants, number of genotypes, distribution of alleles, sample size in case, and control groups.

### Statistical analysis

In our meta-analysis, 5 genetic models as the allelic (C vs. S of *PON2* Ser311Cys gene polymorphism; G vs. A of *PON2* Ala148Gly gene polymorphism), homozygous (CC vs. SS of *PON2* Ser311Cys gene polymorphism; GG vs. AA of *PON2* Ala148Gly gene polymorphism), dominant (CC+SC vs. SS of *PON2* Ser311Cys gene polymorphism; GG+AG vs. AA of *PON2* Ala148Gly gene polymorphism), heterozygous (SC vs. SS of *PON2* Ser311Cys gene polymorphism; AG vs. AA of *PON2* Ala148Gly gene polymorphism) and recessive (CC vs. SS+SC of *PON2* Ser311Cys gene polymorphism; GG vs. AA+AG of *PON2* Ala148Gly gene polymorphism) were performed.

The odds ratio (OR) and their corresponding 95% confidence interval (CI) were employed to assess the association of *PON2* Ser311Cys and Ala148Gly gene polymorphisms with the risk of T2DM. The pooled OR was evaluated by *Z*-test with significance set at *P*-value < 0.05. The test for heterogeneity between studies was performed using the Chi-square based *Q*-test and Higgins *I*^2^ index (ranging from 0 to 100%) (21). If there was significant heterogeneity between studies, the random-effect model using DerSimonian and Laird method was used. Otherwise, the fixed-effect model (Mantel–Haenszel method) was adopted for the meta-analysis. Galbraith plot was used to explore sources of between-study heterogeneity. The potential publication bias was evaluated by Begg's funnel plot and Egger's linear regression test (22, 23). The statistical analyses were performed by using Stata 12.0 software (StataCorp, College Station, TX, USA).

## Results

### Summary of included studies

**Figure 1:**
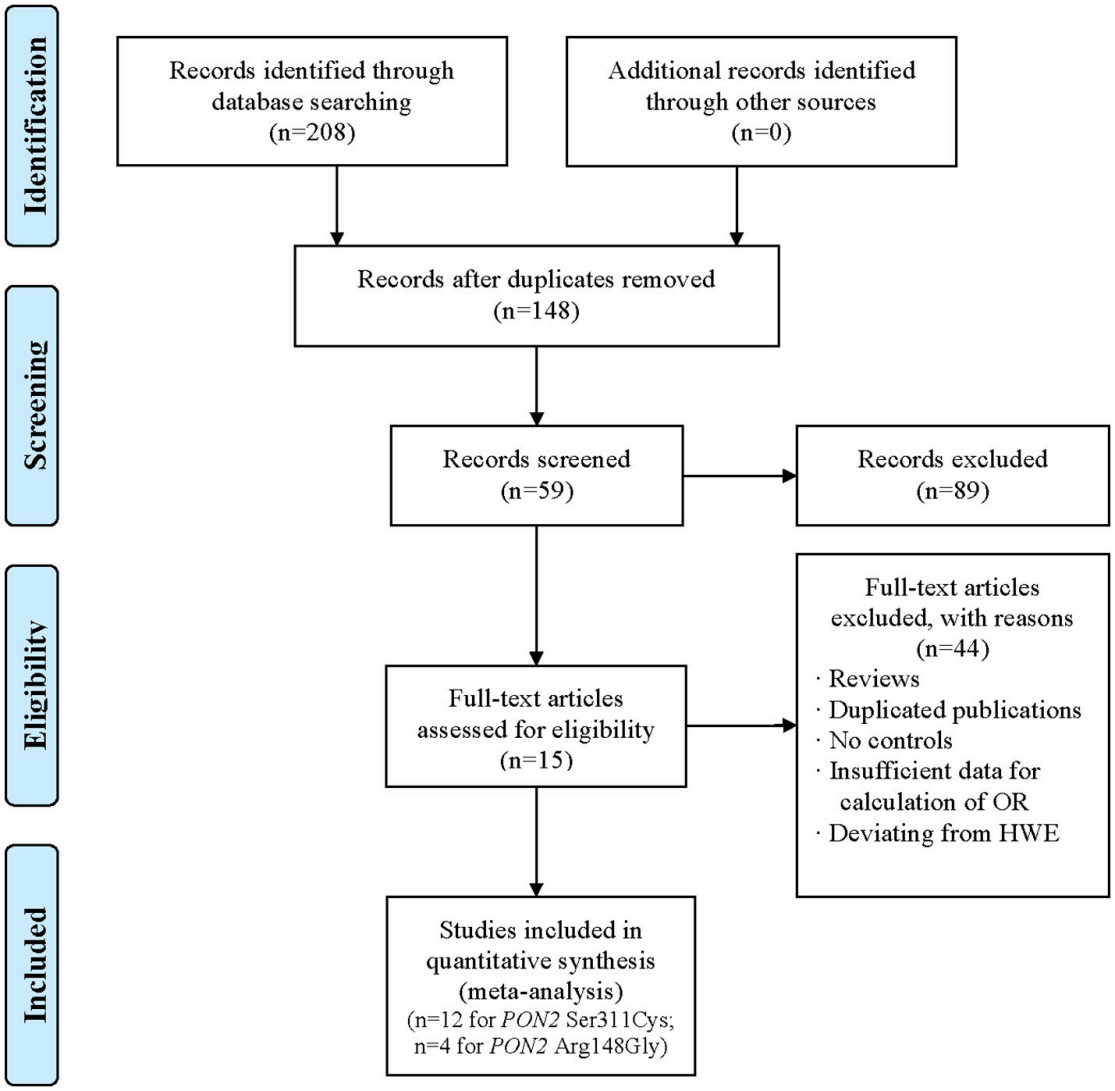
Flow diagram of the selection process of research studies The terms “*n*” in the boxes represent the number of corresponding studies.

In the initial screening, 208 articles were identified and 60 articles were excluded due to duplicate publication. Based on the inclusion criteria for meta-analysis the association of *PON2* Ser311Cys and Arg148Gly polymorphisms with T2DM, 89 articles were excluded after screening the abstract and title, and 44 articles were excluded after screening the full-texts. Finally, 15 articles were included. One article investigated the two polymorphisms in the same population. As a result, a total of 12 eligible studies were included for meta-analysis of *PON2* Ser311Cys polymorphism (15–19, 24–30), and 4 studies for meta-analysis of *PON2* Arg148Gly polymorphism (17, 31–33). Figure 1 illustrated the selection process. Characteristics of the 16 studies on *PON2* Ser311Cys and Arg148Gly polymorphisms and risk of developing T2DM susceptibility were summarized in Table 1. In addition, the HWE-P value in the control group in all studies was also listed in Table 1.

**Table 1:** Characteristics of included studies of the association of *PON2* Ser311Cys and Arg148Gly genetic polymorphisms with type 2 diabetes mellitus.

**References**	**Area^a^**	**Male/Female**		**Age(years)**		**Genotyping method**	**Sample size^b^**	**Genotypes or alleles (case/control)**^**c**^					**HWE-*P*^d^**
		**Case**	**Control**	**Case**	**Control**			**11**	**12**	**22**	**1**	**2**	
***PON2*** **Ser311Cys**													
Wang et al. (24)	Guangdong	50/55	64/53	57.4 ± 10.8	47.2 ± 11.9	PCR-RFLP	105/117	78/85	25/30	2/2	181/200	29/34	0.726
Wang and Chang (25)	Beijing	24/12	29/9	64.8 ± 11.9	70.8 ± 10.8	PCR-RFLP	36/38	12/13	19/17	5/8	43/43	29/33	0.581
Fu and Zhang (26)	Henan	NA	NA	59.9 ± 7.0	61.9 ± 6.8	PCR-RFLP	47/40	17/14	24/17	6/9	58/45	36/35	0.388
Shi et al. (27)	Gansu	14/12	18/12	54.0 ± 12.0	53.0 ± 4.0	PCR-RFLP	26/30	10/11	13/13	3/6	33/35	19/25	0.552
Jiang et al. (28)	Shanxi	20/22	21/24	54.9 ± 10.6	48.5 ± 13.1	TDI-FP	42/45	15/23	16/16	11/6	46/62	38/28	0.253
Sun et al. (17)	Jilin	92/85	50/47	64.5 ± 10.3	62.4 ± 10.9	PCR-RFLP	167/97	102/32	55/39	10/26	259/103	75/91	0.058
Xu et al. (18)	Anhui	166/161	90/94	60.7 ± 8.9	55.1 ± 9.2	PCR-RFLP	327/184	205/123	108/52	14/9	518/298	136/70	0.262
Qu et al. (15)	Mixed	204/230	245/288	49.7 ± 13.2	48.4 ± 8.2	PCR-RFLP	434/533	276/385	151/133	7/15	703/903	165/163	0.396
Chen et al. (29)	Fujian	50/47	55/50	59.9 ± 10.6	58.7 ± 5.7	PCR-RFLP	97/105	71/71	21/28	5/6	163/170	31/40	0.166
Sun et al. (19)	Heilongjiang	79/131	181/138	NA	NA	PCR-RFLP	210/319	134/200	69/95	3/8	337/495	75/111	0.406
Xu and Dai (16)	Qinghai	NA	63/25	NA	72.0 ± 9.6	PCR-RFLP	18/88	5/59	7/25	6/4	17/143	19/33	0.526
Ma et al. (30)	Shanxi	NA	NA	NA	NA	PCR-RFLP	65/70	23/34	32/30	10/6	78/98	52/42	0.864
***PON2*** **Arg148Gly**													
Hao et al. (31)	Fujian	44/34	20/23	53.4 ± 3.2	53.4 ± 3.6	PCR-RFLP	78/43	51/30	24/12	3/1	126/72	30/14	0.876
Feng et al. (32)	Beijing	65/67	67/65	53.2 ± 10.0	53.7 ± 11.2	PCR-RFLP	132/132	81/78	42/40	5/6	204/196	52/52	0.766
Wang and Hu (33)	Beijing	58/47	51/49	59.27±*NA*	59.35±*NA*	PCR-RFLP	105/100	74/70	27/24	4/6	175/164	35/36	0.061
Sun et al. (17)	Jilin	92/85	50/47	64.5 ± 10.3	62.4 ± 10.9	PCR-RFLP	154/97	89/71	54/21	11/5	232/163	76/31	0.056

*HWE, Hardy–Weinberg equilibrium; NA, not available PCR-RFLP, polymerase chain reaction-restriction fragment length polymorphism; TDI—FP, template-directed dye-terminator incorporated with fluorescence polarization detection (TDI-FP)*.

a *Mixed means Beijing and Heinongjiang*.

b *Sample size means the case/control groups*.

c *For the PON2 Ser311Cys, 11: SS, 12: SC, 22: CC; For the PON2 Arg148Gly, 11: AA, 12:AG, 22: GG*.

d *P value for Hardy–Weinberg equilibrium test in controls*.

### Pooled analyses

**Figure 2:**
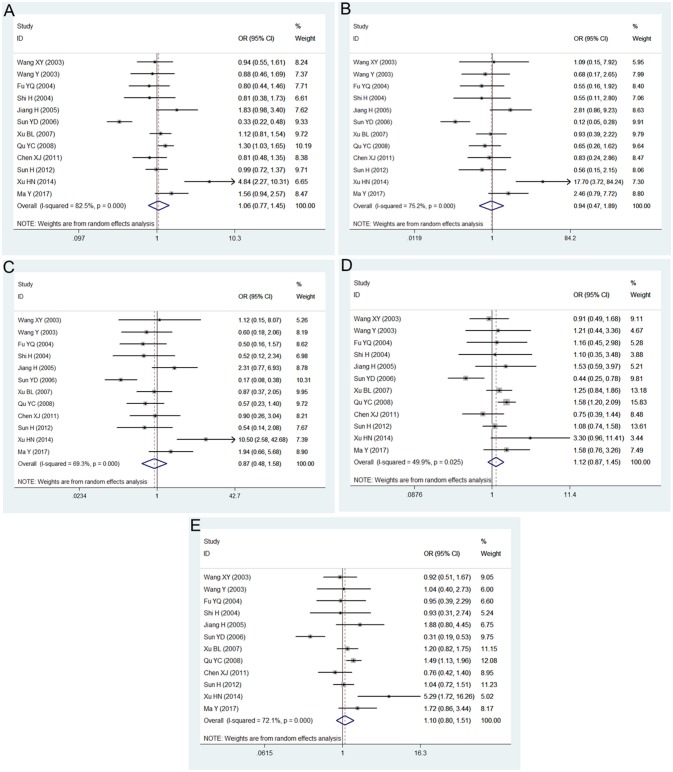
Forest plot of the meta-analysis for association between *PON2* Ser311Cys polymorphism and type 2 diabetes risk under the allelic **(A)**, homozygous **(B)**, recessive **(C)**, heterozygous **(D)**, and dominant **(E)** genetic model.

The main results of this meta-analysis for the association between *PON2* Ser311Cys polymorphism and risk of developing T2DM are shown in Table 2. There was no significant association between the *PON2* Ser311Cys polymorphism and T2DM risk under all genetic models: allelic (OR = 1.06, 95% CI = 0.77–1.45; *P* = 0.721), heterozygous (OR = 1.13, 95% CI = 0.87–1.45; *P* = 0.362), dominant (OR = 1.10, 95% CI = 0.80–1.51; *P* = 0.562), recessive (OR = 0.87, 95% CI = 0.48–1.58; *P* = 0.648), homozygous (OR = 0.94, 95% CI = 0.47–1.89; *P* = 0.865) (Figure 2, Table 2).

**Table 2:** Summary of meta-analysis of association between *PON2* Ser311Cys genetic polymorphism and risk of type 2 diabetes mellitus in the Chinese population.

**Genetic model**	**Pooled analysis**					**Tests of heterogeneity**		**Tests of publication bias**	
	**Pooled OR(95%CI)**	***Z*-value**	***P*-value**	***N***	**Model**	***P*-value**	***I*^2^%**	***P*-value (Egger)**	***P*-value (Begg)**
Allelic genetic model	1.06(0.77–1.45)	0.36	0.721	12	R	< 0.001	82.50	0.824	0.837
Recessive genetic model	0.87(0.48–1.58)	0.46	0.648	12	R	< 0.001	69.30	0.133	0.451
Dominant genetic model	1.10(0.80–1.51)	0.58	0.562	12	R	< 0.001	72.10	0.913	0.537
Homozygous genetic model	0.94(0.47–1.89)	0.17	0.865	12	R	< 0.001	75.20	0.126	0.631
Heterozygous genetic model	1.13(0.87–1.45)	0.91	0.362	12	R	0.025	49.90	0.659	0.631

*CI, confidence interval; N, the number of the studies in the meta-analysis; OR, odds ratio; R, random-effects model; T2DM, type 2 diabetes mellitus; Allelic genetic model, C vs. S, Recessive genetic model, CC vs. SC + CC, Dominant genetic model, CC + SC vs. SS, Homozygous genetic model, CC vs. SS, Heterozygous genetic model, SC vs. SS*.

**Figure 3:**
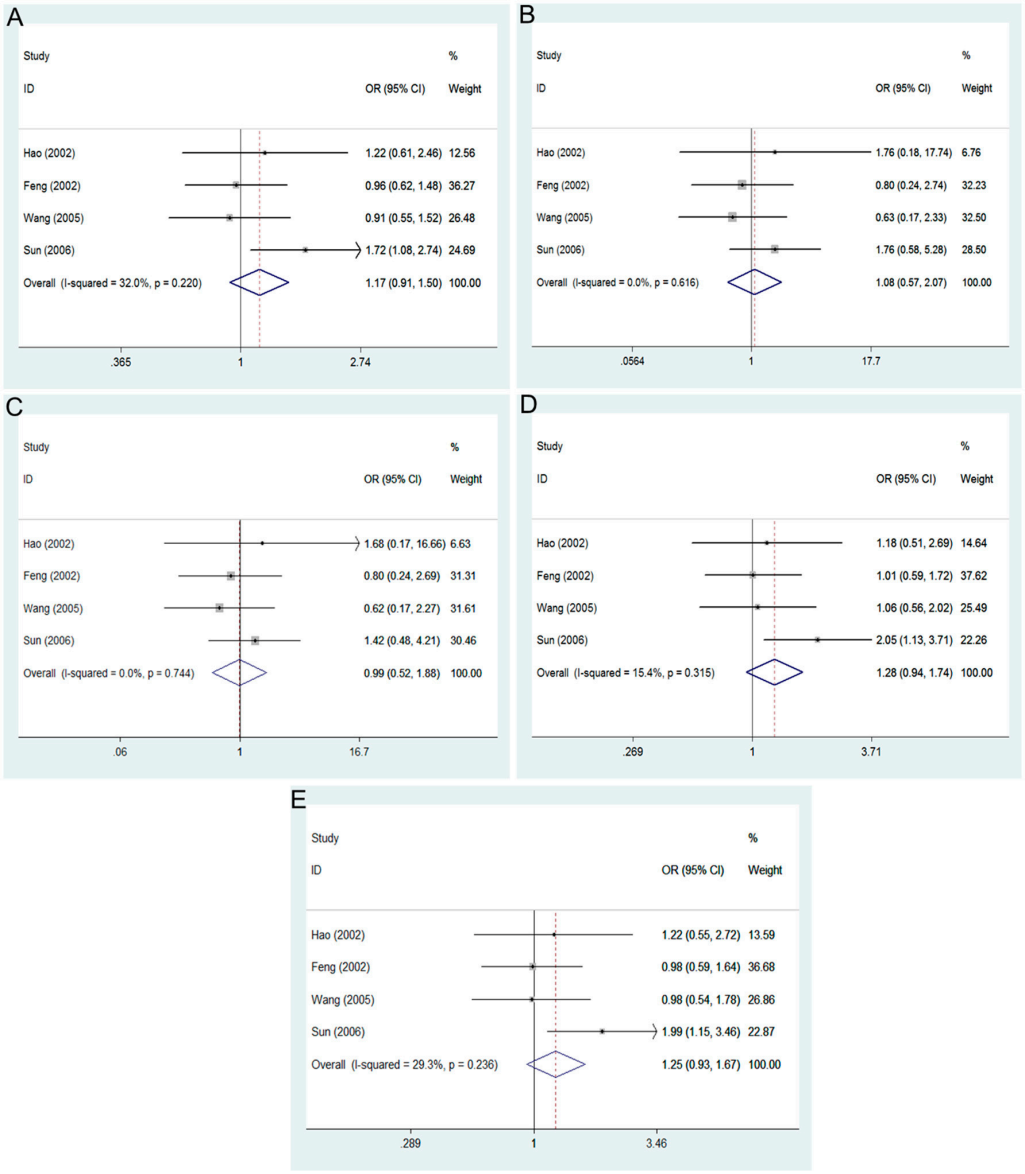
Forest plot of the meta-analysis for association between *PON2* Ala148Gly polymorphism and type 2 diabetes risk under the allelic **(A)**, homozygous **(B)**, recessive **(C)**, heterozygous **(D)**, and dominant **(E)** genetic model.

The main results of this meta-analysis in the association between *PON2* Arg148Gly polymorphism and risk of developing T2DM are listed in Table 3. No significant association between the *PON2* Arg148Gly polymorphism and T2DM risk was also found in all genetic models: allelic (OR = 1.17, 95% CI = 0.91–1.50; *P* = 0.218), heterozygous (OR = 1.28, 95% CI = 0.94–1.74; *P* = 0.117), dominant (OR = 1.25, 95% CI = 0.93–1.67; *P* = 0.142), recessive (OR = 0.99, 95% CI = 0.52–1.88; *P* = 0.973), homozygous (OR = 1.08, 95% CI = 0.57–2.07; *P* = 0.808) (Figure 3, Table 3).

**Table 3:** Summary of meta-analysis of association between *PON2* Arg148Gly genetic polymorphism and risk of type 2 diabetes mellitus in the Chinese population.

**Genetic model**	**Pooled analysis**					**Tests of heterogeneity**	
	**Pooled OR(95%CI)**	***Z*-value**	***P*-value**	***N***	**Model**	***P*-value**	***I*^2^%**
Allelic genetic model	1.17(0.91–1.50)	1.23	0.218	4	F	0.220	32.00
Recessive genetic model	0.99(0.52–1.88)	0.03	0.973	4	F	0.744	0.00
Dominant genetic model	1.25(0.93–1.67)	1.47	0.142	4	F	0.236	29.30
Homozygous genetic model	1.08(0.57–2.07)	0.24	0.808	4	F	0.616	0.00
Heterozygous genetic model	1.28(0.94–1.74)	1.57	0.117	4	F	0.315	15.40

*CI, confidence interval; N, the number of the studies in the meta-analysis; OR, odds ratio; R, random-effects model; T2DM, type 2 diabetes mellitus; Allelic genetic model, G vs. A, Recessive genetic model, GG vs. AG + AA, Dominant genetic model, GG + AG vs. AA, Homozygous genetic model, GG vs. AA, Heterozygous genetic model, AG vs. AA*.

### Sources of heterogeneity

The results of the heterogeneity analysis in *PON2* Ser311Cys polymorphism are summarized in Table 2. There was significant between-study heterogeneity under all genetic models (allelic: *I*^2^ = 82.50%, *P*_heterogeneity_ < 0.001; recessive: *I*^2^ = 69.30%, *P*_heterogeneity_ < 0.001; dominant: *I*^2^ = 72.10%, *P*_heterogeneity_ < 0.001; homozygous: *I*^2^ = 75.20%, *P*_heterogeneity_ < 0.001; heterozygous: *I*^2^ = 49.90%, *P*_heterogeneity_ = 0.025). By contrast, as is shown in Table 3, there was no heterogeneity in the meta-analysis in *PON2* Arg148Gly polymorphism under all five genetic models (allelic: *I*^2^ = 32.00%, *P*_heterogeneity_ = 0.220; recessive: *I*^2^ = 0%, *P*_heterogeneity_ = 0.744; dominant: *I*^2^ = 29.30%, *P*_heterogeneity_ = 0.236; homozygous: *I*^2^ = 0%, *P*_heterogeneity_ = 0.616; heterozygous: *I*^2^ = 15.40%, *P*_heterogeneity_ = 0.315).

**Figure 4:**
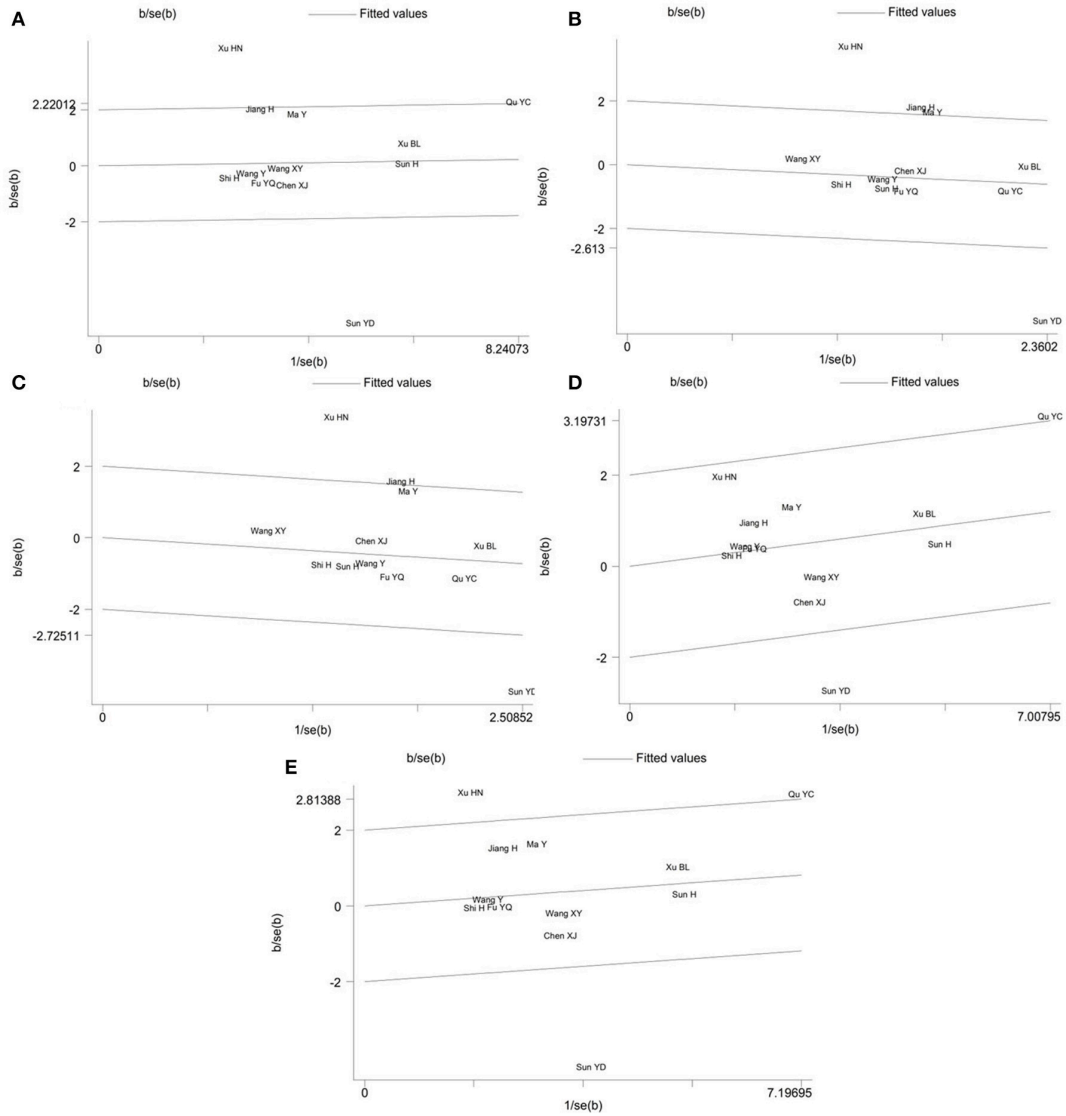
Galbraith plot of the meta-analysis for association between *PON2* Ser311Cys polymorphism and type 2 diabetes risk under the allelic **(A)**, homozygous **(B)**, recessive **(C)**, heterozygous **(D)**, and dominant **(E)** genetic model.

Galbraith plot was performed to detect whether there were outliers which could be the potential sources of between-study heterogeneity in the meta-analysis of *PON2* Ser311Cys polymorphism. The analysis showed that the studies conducted by Sun et al. (17) and Xu and Dai (16) were the outliers under the allelic (Figure 4A), homozygous (Figure 4B) and recessive (Figure 4C) genetic models. For the heterozygous (Figure 4D) and dominant (Figure 4E) genetic models, Galbraith plot analysis indicated that Sun YD and Qu YC's study were the outliers. In addition, Xu HN's study may also contribute to the significant heterogeneity under a dominant genetic model.

After exclusion of these outliers studies from the meta-analysis, the recalculated summary ORs were still insignificant but the between-study heterogeneity significantly decreased in all genetic models: allelic (*I*^2^ = 14%, *P*_heterogeneity_ = 0.314), homozygous (*I*^2^ = 0%, *P*_heterogeneity_ = 0.475), recessive (*I*^2^ = 0%, *P*_heterogeneity_ = 0.512), heterozygous (*I*^2^ = 0%, *P*_heterogeneity_ = 0.706) and dominant (*I*^2^ = 0%, *P*_heterogeneity_ = 0.721) (Table 4).

**Table 4:** Summary of meta-analysis of association between *PON2* Ser311Cys genetic polymorphism and risk of type 2 diabetes mellitus in the Chinese population after omitting the outliers.

**Genetic model**	**Pooled analysis**					**Tests of heterogeneity**		**References**
	**Pooled OR (95%CI)**	***Z*-value**	***P*-value**	***N***	**Model**	***P*-value**	***I*^2^%**	
Allelic genetic model	1.12(0.98–1.28)	1.73	0.084	10	F	0.314	14.00	Sun et al. (17); Xu and Dai (16)
Recessive genetic model	0.86(0.61–1.22)	0.85	0.397	10	F	0.512	0.00	Sun et al. (17); Xu and Dai (16)
Dominant genetic model	1.10(0.91–1.34)	0.97	0.332	9	F	0.721	0.00	Sun et al. (17); Qu et al. (15); Xu and Dai (16)
Homozygous genetic model	0.94(0.65–1.36)	0.32	0.751	10	F	0.475	0.00	Sun et al. (17); Xu and Dai (16)
Heterozygous genetic model	1.15(0.95–1.41)	1.40	0.161	10	F	0.706	0.00	Sun et al. (17); Qu et al. (15)

*CI, confidence interval; F, fixed-effects model; N, the number of the studies in the meta-analysis; OR, odds ratio; T2DM, type 2 diabetes mellitus; Allelic genetic model, C vs. S, Recessive genetic model, CC vs. SC + CC, Dominant genetic model, CC + SC vs. SS, Homozygous genetic model, CC vs. SS, Heterozygous genetic model, SC vs. SS*.

### Publication bias analyses

**Figure 5:**
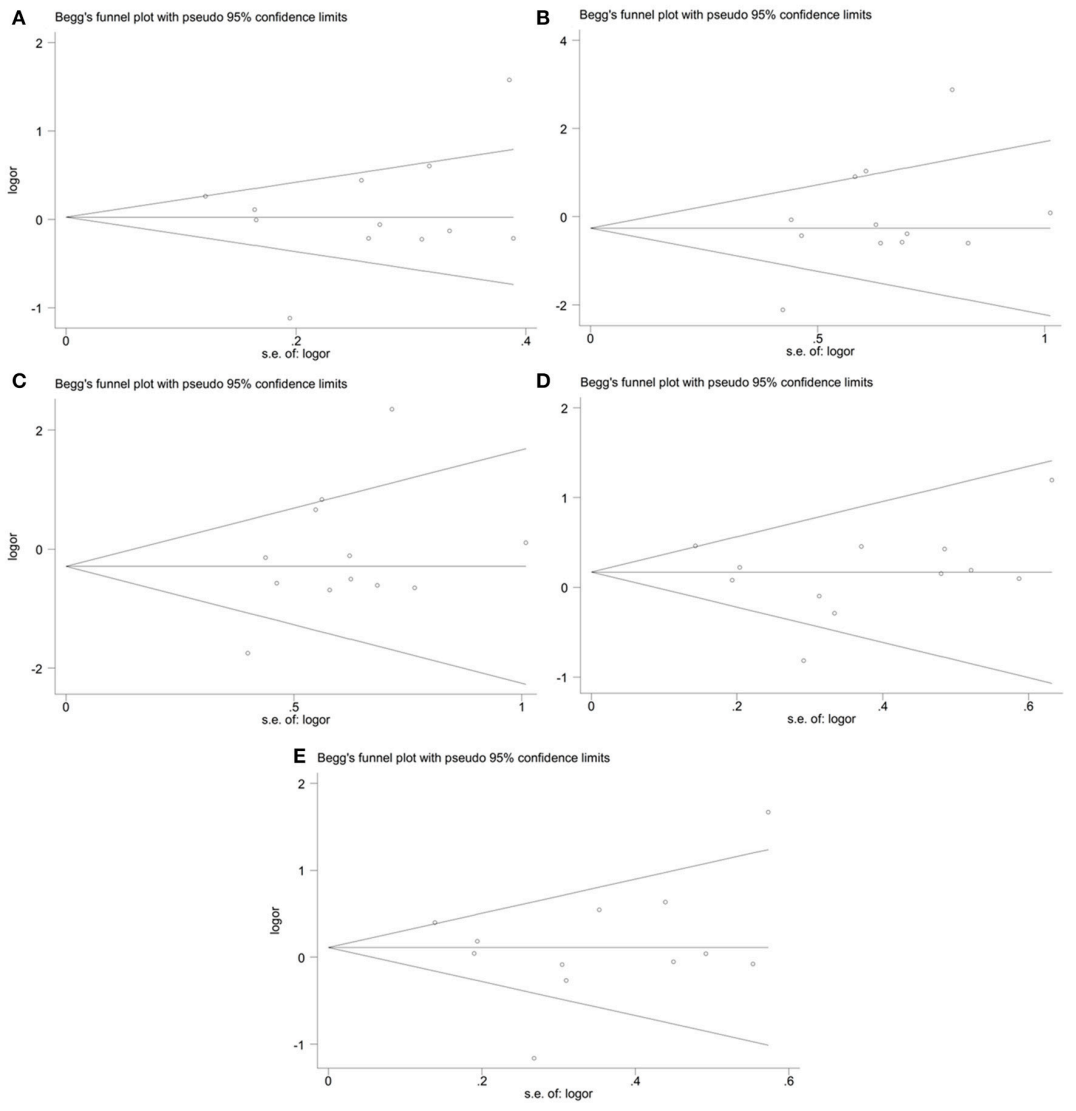
Begg's funnel plot of the meta-analysis for association between *PON2* Ser311Cys polymorphism and type 2 diabetes risk under the allelic **(A)**, homozygous **(B)**, recessive **(C)**, heterozygous **(D)**, and dominant **(E)** genetic model.

The publication bias in the meta-analysis of *PON2* Ser311Cys polymorphism was assessed by Begg's funnel plot (Figure 5, Table 2) and Egger's test (Table 2). The Begg's funnel plot appeared symmetric in all genetic models, with *P* = 0.824 for allelic genetic model (Figure 5A); *P* = 0.126 for homozygous genetic model (Figure 5B); *P* = 0.133 for recessive genetic model (Figure 5C); *P* = 0.659 for heterozygous genetic model (Figure 5D); *P* = 0.913 for dominant genetic model (Figure 5E), suggesting no evidence of publication bias.

Moreover, no evidence of publication bias was also detected by Egger's test (*P* = 0.837 for allelic genetic model; *P* = 0.451 for recessive genetic model; *P* = 0.537 for dominant genetic model; *P* = 0.631 for homozygous genetic model; *P* = 0.631 for heterozygous genetic model).

## Discussion

T2DM is a silent progressive polygenic disease and associated with a number of genetic factors. The role of *PON2* gene in the glycemic control and risk of developing T2DM may be attributed to the widespread tissue expression of PON2, especially the expression in the pancreas. In addition, the expression in the cardiac and skeletal muscle suggests that PON2 could play important roles in the peripheral utilization of glucose (34). Consequently, a large number of researchers have focused on the associations of the *PON2* Ser311Cys and Ala148Gly variations with T2DM risk in the Chinese population. However, the genetic association between the two *PON2* SNPs and the risk of developing 2DM was uncertain owing to conflicting results generated by various independent case-control studies.

To our knowledge, this is the first comprehensive meta-analysis to assess the genetic association between *PON2* Ser311Cys and Ala148Gly polymorphisms and T2DM risk in the Chinese population. Our findings demonstrated that *PON2* Ser311Cys and Ala148Gly genetic polymorphisms were not significantly associated with the risk of developing of T2DM in the Chinese population. The pooled OR and 95% CI were examined with five genetic models including the allelic, homozygous, heterozygous, recessive and dominant, and consistently no significant effects of *PON2* Ser311Cys and Ala148Gly genotypes on T2DM risk were found.

Interestingly, both *PON2* Ser311Cys and Ala148Gly polymorphisms were found not to be associated with the susceptibility of T2DM. The two polymorphisms, Ser311Cys and Ala148Gly, were located at the exon nine and exon five of the *PON2* gene, respectively. By checking the two SNPs (Ser311Cys/rs6954345/rs7493 and Arg148Gly/rs11545942/rs12026) information available for Chinese population in the 1000 genomics database (https://www.ncbi.nlm.nih.gov/variation/tools/1000genomes/), we found that the minor allele frequency of *PON2* Ser311Cys and Ala148Gly were the same each other: 0.20 in Southern Han Chinese and 0.18 in Beijing Han Chinese. In addition, Dasgupta et al. reported that *PON2* Ser311Cys and Ala148Gly polymorphisms were in strong linkage disequilibrium (LD) with each other (*r*^2^ = 0.81) (35). Therefore, strong LD between *PON2* Ser311Cys and Ala148Gly polymorphisms may explain the consistent findings existed in the two *PON2* genetic polymorphisms.

In the meta-analysis of *PON2* Ser311Cys polymorphism, there was substantial between-study heterogeneity under all five genetic models. Therefore, the Galbraith plot was employed to identify the outliers that could be the possible sources of between-study heterogeneity. The Galbraith plot analyses indicated that the studies conducted by Sun et al. (17), Xu and Dai (16), and Qu et al. (15) were the outliers and could largely account for the significant heterogeneity when all eligible studies were pooled into our meta-analysis. Xu HN's study had the largest OR while Sun YD's study had the smallest OR among the included studies. After omitting these outlier studies, the *I*^2^ values immediately decreased to 14% in an allelic genetic model and 0% in other four genetic models, and the *P*-values of the *Q*-test in all genetic models were >0.1. Moreover, the pooled ORs remained statistically insignificant in all genetic models, which demonstrated that our meta-analysis results were stable and reliable.

One previous meta-analysis assessed the association of *PON2* Ser311Cys and Ala148Gly gene polymorphisms with diabetic nephropathy and retinopathy in Caucasian populations (36). However, only three studies included PON2 Ser311Cys and two studies included Ala148Gly in the meta-analysis. The results showed these two *PON2* genetic polymorphisms were not associated with diabetic nephropathy and retinopathy in Caucasians. In addition, numerous meta-analyses have been conducted to determine the association of *PON2* Ser311Cys and Ala148Gly gene polymorphisms with the risk of developing other diseases, such as coronary heart disease (37–39), ischemic stroke (40, 41) and Alzheimer Disease (42). This current meta-analysis only focused on the association of *PON2* Ser311Cys and Ala148Gly gene polymorphisms and the risk of developing T2DM.

Our study has some limitations. First, some of the included studies were based on small sample size, which may have resulted in a decreased power to detect a significant difference in the distribution of genotypes or alleles between cases and controls. Second, the pooled OR is based on the crude OR in the original studies. Because we could not obtain enough raw data from individual studies, the pooled data were not adjusted by potential confounding factors such as gender, age, smoking status, body mass index and waist-hip ratio. Third, T2DM is a polygenic hereditary disorder. The current study only focused on the role of *PON2* genetic polymorphisms in the susceptibility to T2DM. Other susceptibility genes such as *PON1* and *PON3* gene may interact with *PON2* gene by gene-to-gene effect for T2DM (34, 43). Fourth, various environmental factors may be involved in the T2DM risk, the effect of gene-to-environment interactions should be taken into account. For example, synergistic effects between the *PON2* Ala148Gly polymorphism and obesity were found in the risk of T2DM (32). Finally, the current meta-analysis was based on data from candidate gene association studies because the genome-wide association studies (GWAS) data was not collected. Nevertheless, there is no evidence of publication bias assessed by Begg's funnel plot and Egger's test in the meta-analysis.

In conclusion, our meta-analysis confirmed that *PON2* Ser311Cys and Ala148Gly gene polymorphisms did not have a significant association with the risk of developing T2DM in the Chinese population. A well-designed study, with consideration of gene-to-gene and gene-to-environment interactions, should be conducted in the future.

## Author contributions

HR and J-QL conceived and designed the study. J-QL, HR, S-LT, and M-ZL performed the search. HR, S-LT, and J-QL analyzed the data. J-QL, HR, and M-ZL contributed reagents, material, analysis tools. HR, S-LT, J-QL, and HB wrote and review the manuscript. HR and S-LT revised the manuscript. HR and J-QL reference collection, data management, statistical analyses, paper writing, and study design.

### Conflict of interest statement

The authors declare that the research was conducted in the absence of any commercial or financial relationships that could be construed as a potential conflict of interest.
